# Geriatric depression and white matter hyperintensities in Alzheimer’s disease

**DOI:** 10.1192/j.eurpsy.2026.10736

**Published:** 2026-07-31

**Authors:** M.-D. Kim, W.-M. Bahk, B.-H. Yoon, Y.-E. Jung

**Affiliations:** 1 Psychiatry, Jeju National University Hospital, Jeju; 2 Psychiatry, Woo and Bahk Psychiatric Clinic, Seoul; 3 Psychiatry, Naju National Hospital, Naju, Korea, Republic Of

## Abstract

**Introduction:**

White matter hyperintensities (WMHs) in magnetic resonance imaging (MRI) scans are age related but appear to be more prevalent in depressed patients.

**Objectives:**

We aimed to assess whether WMHs are associated with depressive symptoms and physical performance in Alzheimer`s disease(AD)

**Methods:**

One hundred twenty one

AD who visited the Psychiatriy department of Jeju National University Hospital underwent MRI scans. The volume of WMHs was quantified using Lesion Segmentation Toolbox (LST) which allows the performance of accurate brain tissue volume measurements without any kind of manual intervention. After then, for the volume of WMHs, we used a logarithmic transformation to produce normally distributed data. Subjects were assessed for depressive symptoms (GDS-15), cognitive (CERAD-K) and physical (muscle mass index, grip strength, gait speed and timed up and go test) functions. Korean Frailty Index (K-FI) also performed.

**Results:**

WMHs of presumed vascular origin, also referred to as leukoaraiosis, are a very common finding on brain MRI or computed tomography (CT) in older subjects. Most recent studies have shown that WMHs are associated with a host of poor outcomes, including cognitive impairment, depression, gait disturbance and increased risk of death. Thus, it is important to determine the impact of WMHs on mood, physical and cognitive function in AD. In 121 subjects, the mean age was 80.6±7.7.

After analysis, we found that the degree of WMHs was associated with higher depression scores after controlling for age, sex, sum of box and estimated total intracranial volume(p=0.023). Gait speed(p<0.001), timed up and go test(p=0.02), and FI(p=0.002) were significantly associated with the degree of WMHs, though not cognitive function assessed by CERAD-K.

**Conclusions:**

In AD, the degree of WMHs was associated with depressive symptoms as well as gait and balance impairment, though not cognitive function. However, there were further study is required to identify the association between WMHs and other variables.

**Disclosure of Interest:**

None Declared

